# Supplementary material for: The Genetic Background Is Shaping Cecal Enlargement in the Absence of Intestinal Microbiota
Source: Nutrients. 2023 Jan 26;15(3):636. doi: 10.3390/nu15030636 (PMC9921660; doi:10.3390/nu15030636)
Supplement: Supplementary file 1 [file nutrients-15-00636-s001.zip › Supplementary_data_Bolsega-et-al-revised.pdf]

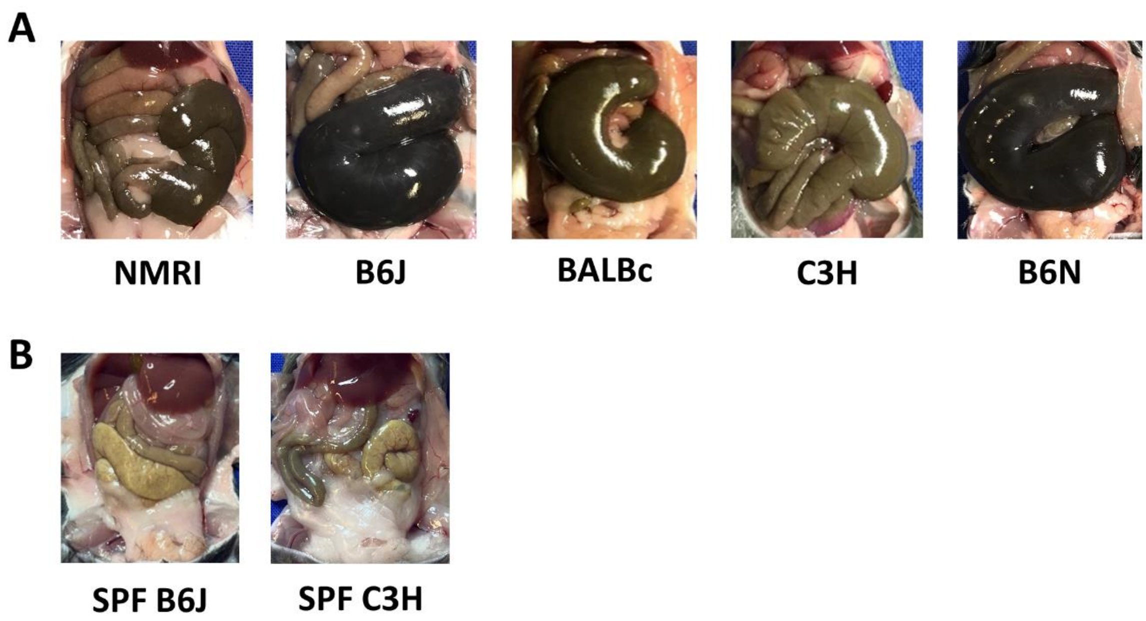

Supplementary Figure S1: Representative photographs of the abdominal cavity with cecum in A) five GF strains (NMRI, B6J, BALBc, C3H and B6N; n=10) and B) complex microbiota colonized B6J and C3H mice (n=10).

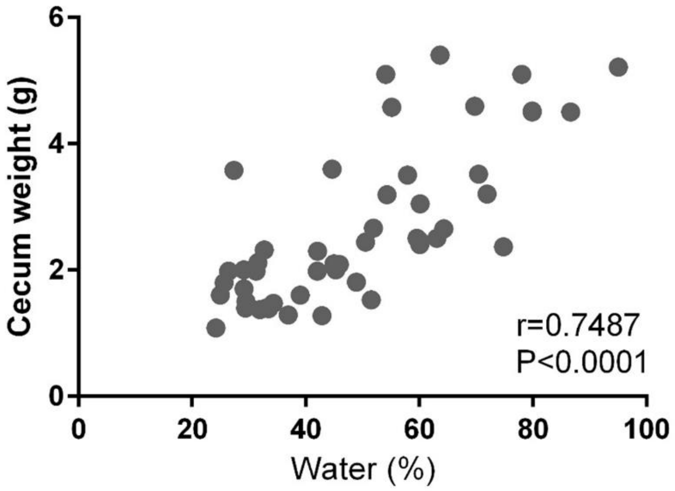

Supplementary Figure S2: Pearson correlation calculations between water content (%) and cecum weight (g).

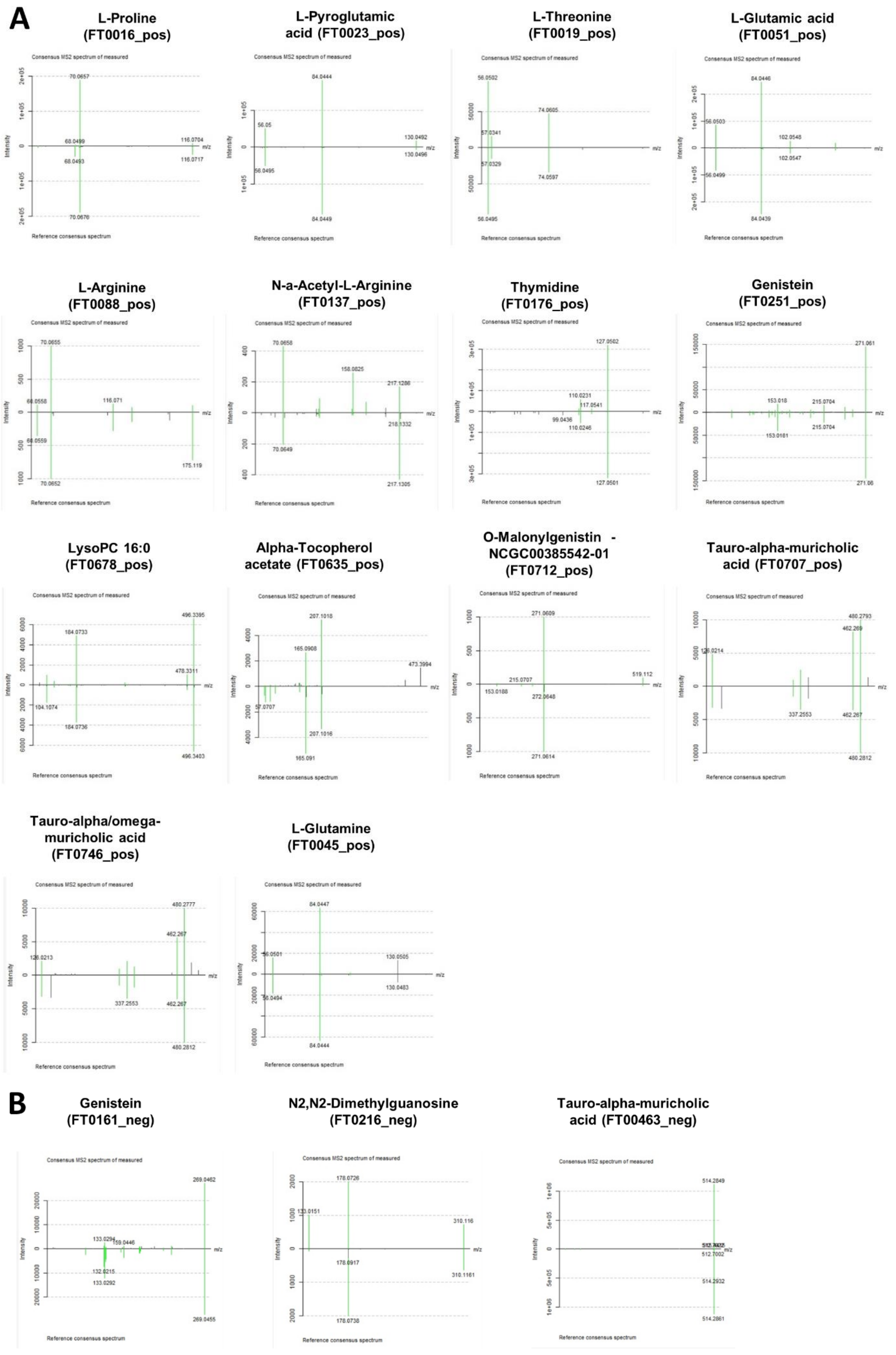

Supplementary Figure S3: MS2 annotation spectra of significantly upregulated metabolic features in cecal content of GF B6J mice detected in A) positive ion mode and B) negative ion mode.

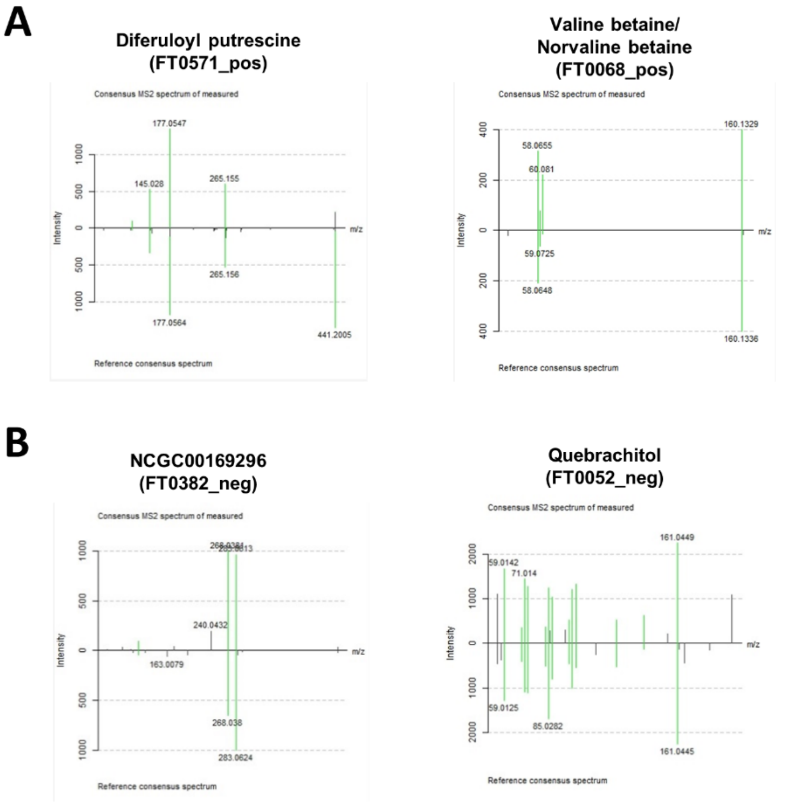

Supplementary Figure S4: MS2 annotation spectra of significantly upregulated metabolic features in cecal content of GF C3H mice detected in A) positive ion mode and B) negative ion mode.

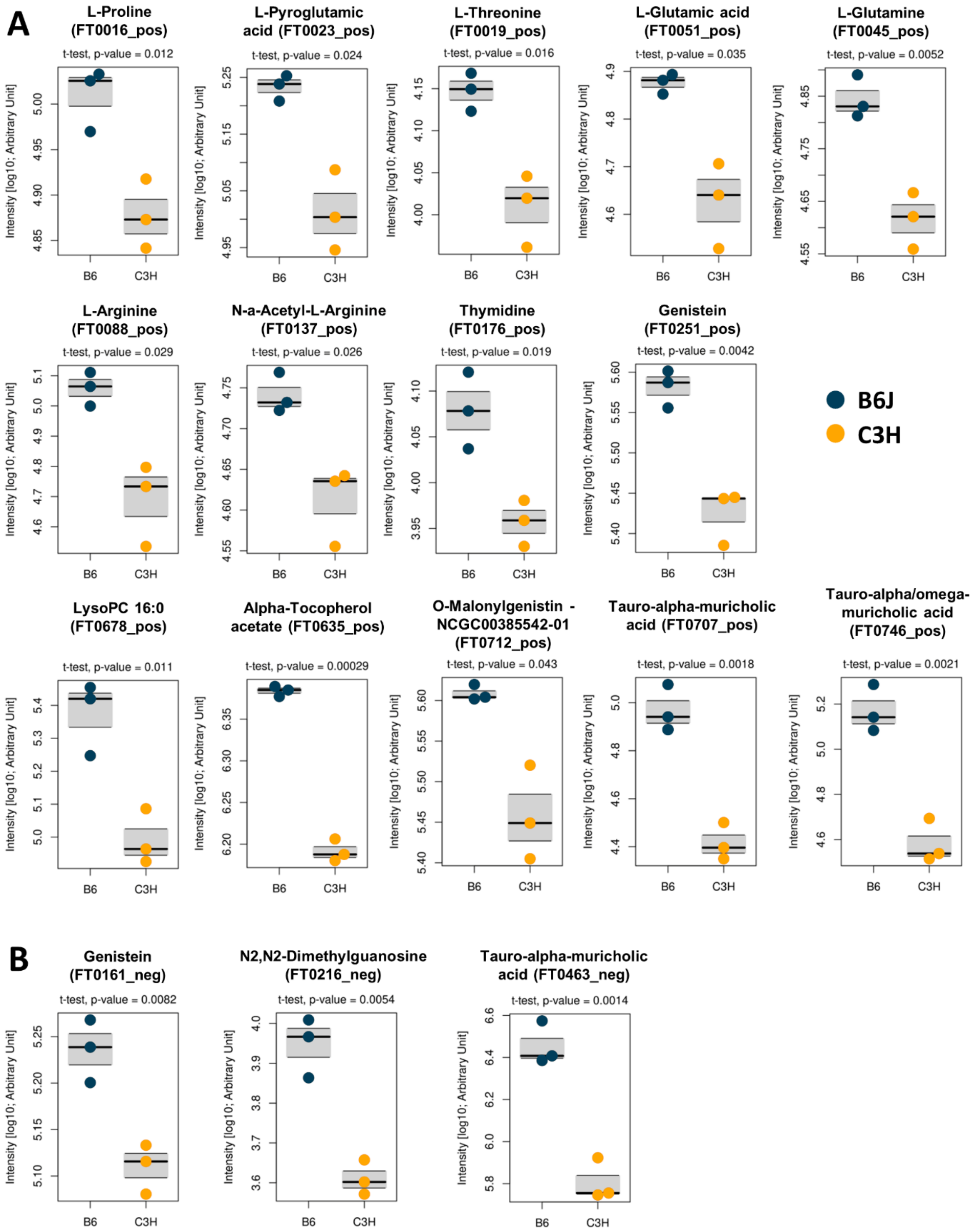

Supplementary Figure S5: Expression profiles of significantly upregulated metabolites in cecal content of GF B6J in comparison with GF C3H mice: A) metabolites measured in positive ion mode and B) metabolites measured in negative ion mode.

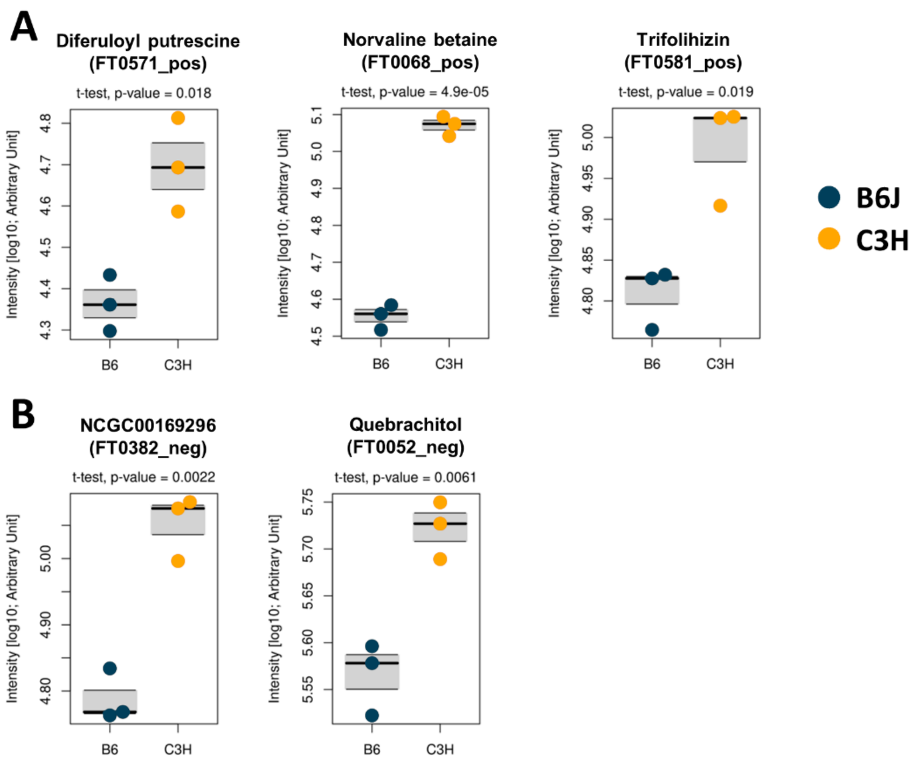

Supplementary Figure S6: Expression profiles of significantly upregulated metabolites in cecal content of GF C3H mice in comparison with GF B6J mice: A) metabolites measured in positive ion mode and B) metabolites measured in negative ion mode.

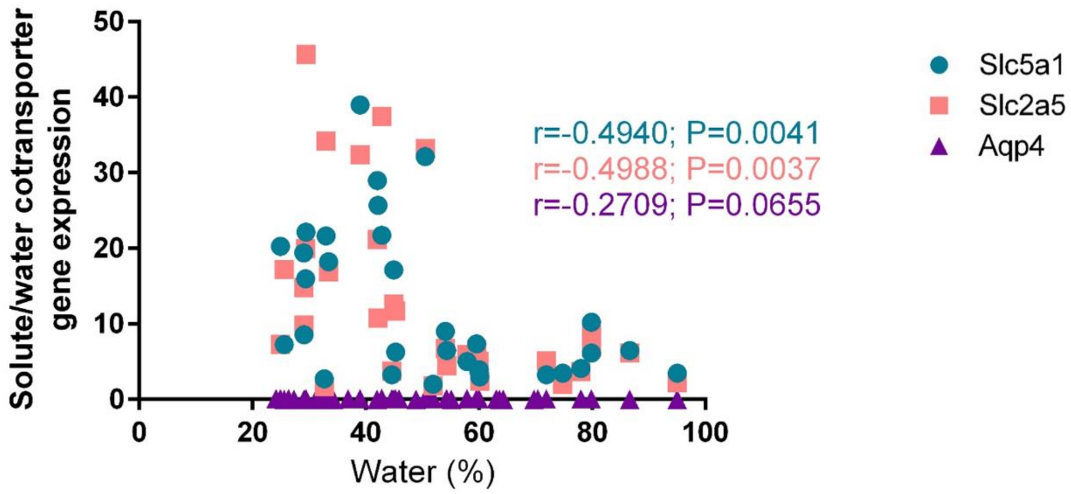

Supplementary Figure S7: Pearson correlation calculations between water content (%) and gene expression of solute/water cotransporters (*Slc5a1, Slc2a5*) and water channel aquaporin *Aqp4.*
